# Robust Pedestrian Dead Reckoning Based on MEMS-IMU for Smartphones

**DOI:** 10.3390/s18051391

**Published:** 2018-05-01

**Authors:** Jian Kuang, Xiaoji Niu, Xingeng Chen

**Affiliations:** GNSS Research Center, Wuhan University, 129 Luoyu Road, Wuhan 430079, China; kuang@whu.edu.cn (J.K.); xingengchen@whu.edu.cn (X.C.)

**Keywords:** SINS, PDR, MEMS-IMU, mobile devices, indoor positioning

## Abstract

This paper proposes a pedestrian dead reckoning (PDR) algorithm based on the strap-down inertial navigation system (SINS) using the gyros, accelerometers, and magnetometers on smartphones. In addition to using a gravity vector, magnetic field vector, and quasi-static attitude, this algorithm employs a gait model and motion constraint to provide pseudo-measurements (i.e., three-dimensional velocity and two-dimensional position increment) instead of using only pseudo-velocity measurement for a more robust PDR algorithm. Several walking tests show that the advanced algorithm can maintain good position estimation compare to the existing SINS-based PDR method in the four basic smartphone positions, i.e., handheld, calling near the ear, swaying in the hand, and in a pants pocket. In addition, we analyze the navigation performance difference between the advanced algorithm and the existing gait-model-based PDR algorithm from three aspects, i.e., heading estimation, position estimation, and step detection failure, in the four basic phone positions. Test results show that the proposed algorithm achieves better position estimation when a pedestrian holds a smartphone in a swaying hand and step detection is unsuccessful.

## 1. Introduction

The rapid development of location-based services (LBSs) has resulted in great convenience in people’s daily lives: personal localization and navigation, and personal tracking and monitoring [1]. A precise location is necessary to provide a high-quality LBS, and the global navigation satellite system (GNSS) can provide accurate locations for pedestrians in the outside open-sky environment. However, the GNSS faces the problems of signal blockage and multipath in urban areas and other challenging environments (e.g., indoor environments). Therefore, indoor localization technology is flourishing, and many different techniques have been designed and developed for tracking pedestrians’ positions when in indoor environments, such as Wi-Fi [2,3], Bluetooth/iBeacon [4,5], radio frequency identification (RFID) [6,7], near-field communication (NFC) [8], ultra-wideband (UWB) [9], magnetic matching [10,11], and inertial-sensor-based [12].

Owing to the many kinds of application requirements and the large cost difference, all of the abovementioned indoor localization methods are at different levels of development. Currently, smartphone-based Wi-Fi indoor localization technology has become the most popular method due to its low cost and the worldwide availability of Wi-Fi access points in the consumer market [13,14]. Wi-Fi signals, however, have poor stability in complex indoor environments, and can be blocked by the human body, as is the case with all other radio-frequency-signal-based indoor location methods. Mobile devices with a built-in micro-electro-mechanical system (MEMS) inertial measurement unit (or IMU, consisting of tri-gyroscopes and tri-accelerometers) can provide autonomous solutions for tracking pedestrians in different types of environments. Thus, they are widely used to overcome the limitations of Wi-Fi signal fluctuations and blockage [3,15].

Two typical IMU-based pedestrian navigation algorithms have been advanced in previous work: pedestrian-gait-model-based (i.e., PDR) and inertial-navigation-system-based (i.e., INS-based). PDR propagates the users’ position following four critical procedures: step detection and step count, step length estimation, moving heading estimation and two-dimensional (2D) position calculation [16,17]. A step detection and step count algorithm is the basic component for checking pedestrian walking behavior, including peak detection [18], zero-crossing [19], auto-correlation [20], dynamic time warp [21], etc. Moreover, a peak detection algorithm is the optimal option for step counting regardless of smartphone placement [22]. After successfully detecting a new step, a step length model (e.g., the Weiberg model and linear model) is used to estimate the moving distance of pedestrians [12,16,23]. In general, heading error causes larger position error than the other components, so an excellent heading-determining method is the key for improving PDR navigation performance. The original PDR algorithm assumes that the output of the vertical gyros only obtains heading changes (i.e., roll and pitch are 0°) [24]. Actually, this assumption will not be satisfied. Thus, an enhanced PDR (E-PDR) algorithm has been designed for improving the accuracy of pedestrian heading angle inspired by the attitude heading reference system (AHRS) [25,26,27]. The AHRS provides a robust and high-precision heading for PDR by fusing tri-accelerometer, tri-gyroscope, and tri-magnetometer measurements. Accelerometers have a good ability to find the leveling angle for perceiving Earth’s gravity, and magnetometers provide heading angles by measuring the environmental magnetic field vector. Although the magnetic field is susceptible to magnetic anomalies, a relative heading change calculated by magnetic measurements in a quasi-static magnetic field is still available for improving heading accuracy [28,29,30,31,32]. 

Another alternative strategy is strap-down-INS-based, which determines the pedestrians’ 3D position, velocity, and attitude by integrating the raw data of tri-accelerometer and tri-gyroscope. Compared with PDR, INS can provide more high-frequency and richer navigation information. However, the position error of INS increases rapidly over time due to the low quality of the MEMS IMU. In order to satisfy the demand of pedestrian navigation applications, some pseudo-observations derived from the kinetic model of humanity are utilized as measurement information for improving INS. In addition, a foot-mounted INS (FM-INS) puts an IMU on the foot (such as toes, instep, or heel) is a typical application case. It is based on the rational idea that movement speed should be zero when a pedestrian’s foot contacts the ground, and zero-velocity update technology (ZUPT) is used to reduce velocity error and can greatly improve the accuracy of position estimation [33]. However, the ZUPT strategy cannot suppress the accumulation of heading errors. In order to mitigate this, several authors have proposed some techniques for estimating gyroscope bias, such as zero angular rate update (ZARU) [34], linear trajectory with unchanged heading update [35], building dominant direction update [36], and magnetic angular rate update (MARU) [37]. At present, the best FM-INS that we can find in the literature can achieve position estimation accuracy in 0.3% of the entire travel distance [38]. However, users need to provide additional inertial sensors as the platform for a FM-INS implementation compared to smartphone-based solutions, and it is necessary to add special protection (e.g., shockproofing and waterproofing) to protect the sensor from damage. Therefore, it is very difficult to implement a FM-INS for wide use in the consumer market.

Owing to the unprecedented growth of smartphone ownership and to the fact that smartphones have a large number of built-in sensors, smartphone-based PDR solutions are generally sensor-available and scalable [39]. However, smartphone-based PDR solutions still present the following issues: (1) smartphone built-in sensors are generally of poor quality [28,29,30,31,32,40], and (2) the misalignment angle between the smartphone heading and a user’s walking direction cannot be assumed to be known [41,42,43,44,45]. E-PDR is a typical solution that overcomes the first issue, but the second issue remains an open problem since some misalignment angle estimation methods have been proposed [41,42,43,44,45]. Inspired by PDR and a FM-INS, researchers have tried to keep the rich navigation information of an INS along with the same navigation position estimation accuracy as E-PDR. The next goal is to quickly and accurately estimate the misalignment angle using the high-precision result of an INS estimation in a short amount of time.

However, due to the poor quality of current smartphone built-in sensors, whether a C-INS has the same navigation performance as E-PDR is still an open question. Researchers have applied the forward walking velocity from the gait model and motion constraint as a 3D velocity update to improve INS velocity (called C-INS) [46]. “Motion constraint” means that the lateral and vertical speeds are zero, which is based on the fact that a moving platform cannot skid or jump. The test results show that a C-INS can provide the same position estimation as E-PDR when an IMU was fixed at the waist. Similarly, other workers [15,24,47] also conducted numerous experiments to verify the performance of C-INS. However, their test results used the original PDR solution (i.e., vertical gyros only obtain heading changes) as the object of comparison, so they cannot be used to illustrate the navigation performance difference between E-PDR and C-INS. The algorithm designed in Ref. [48] implements an E-PDR and a C-INS, using the position calculated by PDR as the position update to improve the INS position. The test results presented in [48] did not provide more details about smartphone placement, and only showed some simple results. Therefore, based on our limited investigation, a navigation performance analysis of the C-INS and a comprehensive performance comparison between C-INS and E-PDR are not available.

In this paper, we focus on the construction of a robust C-INS and provide a complexity comparison between C-INS and E-PDR. In addition to using the gravity vector, magnetic field vector, and quasi-static attitude, the proposed algorithm employs a gait model and motion constraint to provide pseudo-measurements (i.e., 3D velocity and 2D position increment) instead of using only pseudo-velocity measurements for a more robust PDR algorithm. Several walking tests are designed to compare the position estimation performance in the four basic smartphone positions, i.e., handheld, calling near the ear, swaying with the hand, and in a pants pocket, between the proposed C-INS and existing INS-based PDR solutions. We also analyze the performance difference between the proposed C-INS and the existing gait-model-based PDR algorithm from the aspects of heading estimation, position estimation, and step detection failure in the four basic phone positions.

The rest of the paper is organized as follows: in Section 2, the system overview, a simple INS algorithm, a designed extended Kalman filter (EKF) are described in detail, and multi-level measurement equations are presented. The experimental results are described in Section 3. Finally, conclusions and planned future work are summarized in Section 4.

## 2. Materials and Methods

**Figure 1:**
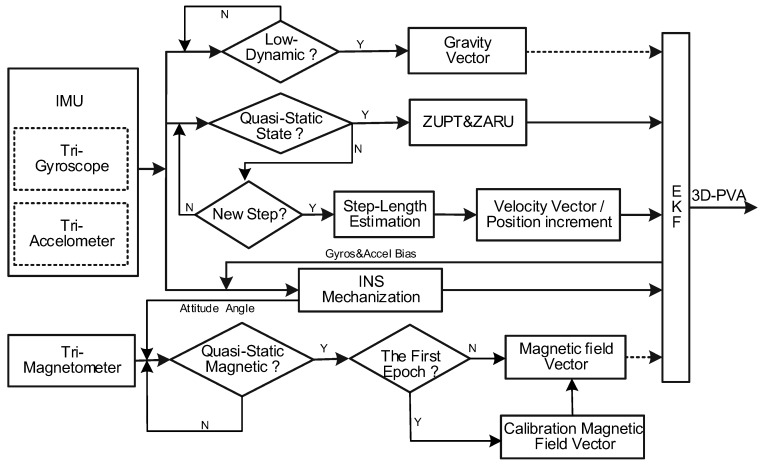
Detailed C-INS solution process.

An overview of the advanced C-INS solution on a smartphone is illustrated in Figure 1. In the traditional PDR method (i.e., E-PDR), raw data from MEMS IMU are used for step detection, step length estimation, and PDR mechanization to estimate the user’s position. In the advanced C-INS algorithm, tri-gyroscope and tri-accelerometer are used to set up the INS mechanization, detect the motion states (e.g., quasi-static, low-dynamic, quasi-static magnetic field, and step event detection), and set up the C-INS mechanization. In Figure 1, if quasi-static state detection is successful, ZUPT is used for the velocity update of the INS to reduce the velocity error; ZARU also applies zero heading rate as the heading update for the INS to improve heading accuracy. However, quasi-static state detection is unsuccessful and step detection is successful; step length estimation is executed to calculate the current step length. Next, the forward velocity and position increment are derived from the estimated step length and estimated heading. The forward velocity works as a velocity update and position increment works as a position update for the INS to improve position estimation. Furthermore, if the low-dynamic detection is successful, the gravity vector is used for roll angle and pitch angle updates for the INS to reduce the leveling angle error. In addition, if the quasi-static magnetic field detection is successful, the magnetic field vector observations at the first epoch are utilized to calibrate the local magnetic field. Next, magnetic vector measurements are used to calculate heading changes for the INS to improve heading estimation.

### 2.1. INS Mechanization

INS mechanization algorithms have been widely developed in strap-down inertial navigation. The main idea is that the current position, velocity, and attitude of a moving object can be obtained by integrating acceleration twice and angular rate once after knowing the initial navigation state (i.e., 3D position, 3D velocity, and 3D attitude). Owing to the low quality of the MEMS-IMU built-in mobile devices, some small error correction terms (i.e., rotation of the Earth) are omitted in the proposed algorithm because those error correction terms are absorbed by sensor noise and only result in a slight improvement of navigation performance. The simplified form of discrete INS mechanization algorithm equations in the navigation coordinate system (i.e., n-frame) is given as follows [33,49]:

(1) $$\left(\begin{array}{c} p_{k}^{n}\\ v_{k}^{n}\\ C_{b,k}^{n} \end{array}\right)=\left(\begin{array}{c} p_{k-1}^{n}+v_{k}^{n}\Delta t\\ v_{k-1}^{n}+\left[C_{b,k}^{n}\left({\tilde{f}}_{k}^{b}-b_{a}\right)-g^{n}\right]\Delta t\\ C_{b,k-1}^{n}+C_{b,k-1}^{n}\Omega \left[\left({\tilde{\omega }}_{k}^{b}-b_{g}\right)\Delta t\right] \end{array}\right)$$

where $p^{n}$ and $v^{n}$ represent the position vector and the velocity vector in the *n*-frame, respectively, $C_{b}^{n}$ the transformation matrix from the *b*-frame (i.e., the body coordinate system) to the *n*-frame, $g^{n}={\left[\begin{array}{ccc} 0 & 0 & -g \end{array}\right]}^{T}$ the Earth gravity vector, ${\tilde{f}}^{b}$ and $b_{a}$ the acceleration measurement vector and the bias vector of the tri-accelerometer, respectively, ${\tilde{\omega }}^{b}$ and $b_{g}$ the angle rate measurement vector and bias vector of the tri-gyroscope, respectively, $\Delta t=t_{k}-t_{k-1}$ the time interval between the *(k−1)*-th and *k*-th epoch, and Ω[·] the cross-product form of a vector.

### 2.2. System Model

As described in Section 2.1, tri-gyroscope measurements are integrated to obtain the attitude angle, and tri-accelerometer measurements are converted from the *b*-frame to the *n*-frame by using the attitude information. Finally, the position is obtained by integrating the acceleration twice. We find that gyro bias introduces a third-order error in position and an accelerometer bias introduces a second-order error in position. Therefore, a low-grade MEMS IMU, which usually has large acceleration and gyroscopes biases, will cause rapid accumulation of position error.

In order to reduce the position drift of a MEMS IMU-based positioning system, an EKF is usually used to fuse multi-measurement information to improve the availability and accuracy of the positioning system. The state variables are usually defined as [33]:

(2) $$\delta x={\left[\begin{array}{ccccc} {\left(\delta p^{n}\right)}_{1\times 3} & {\left(\delta v^{n}\right)}_{1\times 3} & \Psi_{1\times 3} & {\left(b_{g}\right)}_{1\times 3} & {\left(b_{a}\right)}_{1\times 3} \end{array}\right]}^{T}$$

where $\delta p^{n}$, $\delta v^{n}$ and Ψ are the error vector of position, velocity and attitude in the *n*-frame, respectively. $b_{g}$, $b_{a}$ are the bias vector of tri-gyroscope and tri-accelerometer, respectively. The discrete linearization of the system error model can be expressed as follows:

(3) $$\{\begin{array}{c} \delta x_{k|k-1}=\Phi_{k-1}\delta x_{k-1|k-1}+w_{k}\\ \delta z_{k}=H_{k}\delta x_{k|k-1}+v_{k} \end{array}$$

where the subscripts *k−1* and *k* represent the epoch, $\delta x_{k-1|k-1}$ the previous error state vector, $\delta x_{k|k-1}$ the predicted error state vector, $\delta z_{k}$ the measurement misclosure vector, $H_{k}$ the design matrix, $w_{k}$, $v_{k}$ the process noise and measurement noise, respectively, and $\Phi_{k-1}$ the 15 × 15 state transition matrix:

(4) $$\Phi_{k}=\left[\begin{array}{ccccc} I_{3\times 3} & I_{3\times 3}\cdot \Delta t & 0_{3\times 3}^{} & 0_{3\times 3}^{} & 0_{3\times 3}^{}\\ 0_{3\times 3}^{} & I_{3\times 3} & \left(f_{k}^{n}\times \right)\cdot \Delta t & 0_{3\times 3}^{} & C_{b,k}^{n}\cdot \Delta t\\ 0_{3\times 3}^{} & 0_{3\times 3}^{} & I_{3\times 3} & -C_{b,k}^{n}\cdot \Delta t & 0_{3\times 3}^{}\\ 0_{3\times 3}^{} & 0_{3\times 3}^{} & 0_{3\times 3}^{} & I_{3\times 3} & 0_{3\times 3}^{}\\ 0_{3\times 3}^{} & 0_{3\times 3}^{} & 0_{3\times 3}^{} & 0_{3\times 3}^{} & I_{3\times 3} \end{array}\right]$$

where Δt is the time interval between two adjacent epochs. $f_{k}^{n}$ represents the acceleration measurement vector in the *n*-frame. More details of the EKF implementation can be found in Ref. [14].

### 2.3. Multi-Level Measurements

**Figure 2:**
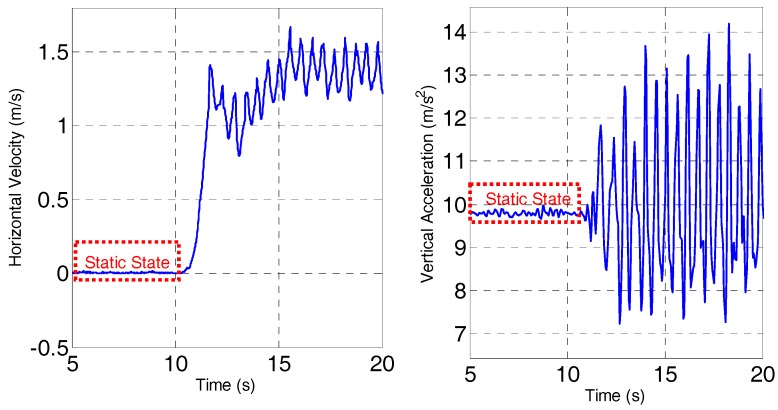
Horizontal velocity (**left**) and vertical acceleration (**right**) during static state and walking.

Analysis of the human gait shows that the basic pattern of human motion maintains a static state without moving or a cyclical, repeatable walk gait, and is remarkably consistent between individuals, as shown in Figure 2. Therefore, we use a pseudo-velocity measurement in which the zero velocity and horizontal moving velocity derived from a human gait are used to improve velocity accuracy. Furthermore, we exploit the complementary characteristics of gyro, accelerometer, and magnetic measurements to ensure the attitude accuracy inspired by the idea of AHRS.

#### 2.3.1. Quasi-State Update

The static state is a very important period for determining the attitude of sensors and calibrating the bias of the tri-gyroscope and tri-accelerometer. A typical assumption for a static state is that the velocity is zero (i.e., ZUPT) and any attitude change sensed by the gyros should be caused by gyro bias (i.e., ZARU). In general, there is no strict static state in pedestrian navigation for human interference. Therefore, we use a quasi-static period that is a dynamically small enough alternative; thus, acceleration and gyro output are employed to detect the quasi-static period in this paper [50]:

(5) $$\frac{1}{N}\sum_{k=1}^{N}(\frac{{\left\|f^{k}-\bar{f}\right\|}^{2}}{\sigma_{f}^{2}}+\frac{{\left\|\omega^{k}\right\|}^{2}}{\sigma_{\omega }^{2}})<\gamma$$

where *N* represents static state detection window length, $f^{k}$ and $\omega^{k}$ the output vectors of tri-accelerometer and tri-gyroscope at epoch *k*, $\bar{f}$ the mean value vector of acceleration, $\sigma_{f}$ and $\sigma_{\omega }$ the sensor noise of accelerometer and gyros, respectively, and γ the detection threshold. When determining the pedestrians in quasi-static state, the velocity observation equation in the *n*-frame for ZUPT is given by:

(6) $$\delta z_{v}={\hat{v}}_{ins}^{n}-v_{meas}^{n}=\delta v^{n}+n_{v}$$

where ${\hat{v}}_{ins}^{n}$ represents the computed velocity vector by INS mechanization in the *n*-frame, $v_{meas}^{n}={\left[\begin{array}{ccc} 0 & 0 & 0 \end{array}\right]}^{T}$ the pseudo-velocity measurements, and $n_{v}$ the measurement noise.

Moreover, the heading observation equation in the *n*-frame for ZARU is given by:

(7) $$\delta z_{\psi }={\hat{\psi }}_{ins}-\psi_{ref}=\delta \psi +n_{\psi }=\left[\begin{array}{ccc} \frac{\partial \psi }{\partial \phi_{x}} & \frac{\partial \psi }{\partial \phi_{y}} & \frac{\partial \psi }{\partial \phi_{z}} \end{array}\right]\phi +n_{\psi }$$

where ${\hat{\psi }}_{ins}$ represents the computed heading angle from INS mechanization, $\psi_{ref}$ the pseudo-heading observations obtained by storing the heading angle at the first epoch of the quasi-static state period, and $n_{\psi }$ the measurement noise. $\left[\begin{array}{ccc} \frac{\partial \psi }{\partial \phi_{x}} & \frac{\partial \psi }{\partial \phi_{y}} & \frac{\partial \psi }{\partial \phi_{z}} \end{array}\right]$ represents the heading design matrix, the detailed equation of which can be found in [49].

#### 2.3.2. Gait Model Update

The basic assumption of typical PDR is that pedestrians, in general, walk along the direction that they are facing. We can reasonably believe that there is only the speed of the forward direction when pedestrians walk in normal mode, and that lateral and vertical speed are almost zero. Based on this basic condition, a ”step detection” algorithm and a “step length estimation” empirical model are used to obtain the average speed between two adjacent steps in the “walk forward” state. We employ the peak detection algorithm to detect a pedestrian step due to its lower amount of computation and higher accuracy [22]. The Weiberg model is utilized to estimate pedestrian step length for less-estimated parameters, which assumes that the step length is proportional to the vertical movement of the human hip. The difference between the maximum and minimum value of the vertical acceleration in each step period is used to calculate movement distance. The equation for step length estimation is expressed as [22]:

(8) $$SL=K\cdot \sqrt[{4}]{a_{z\_max}-a_{z\_min}}$$

where *K* represents the scale factor of the step length, $a_{z-max}$ and $a_{z-min}$ the maximum and minimum value of the vertical acceleration in one step period, respectively. Thus, the mean value of horizontal moving velocity between adjacent two steps in the pedestrian coordinate system (*l*-frame) can be expressed as follows [15]:

(9) $${\tilde{v}}^{l}={\left[\begin{array}{ccc} SL/\Delta t & 0 & 0 \end{array}\right]}^{T}$$

The computed velocity between adjacent two steps in the *l*-frame can be expressed as

(10) $$\begin{array}{ll} {\hat{v}}^{l}\approx & C_{b}^{l}{\hat{C}}_{n}^{b}{\hat{v}}^{n}\\ & \approx C_{b}^{l}C_{n}^{b}\left(I+\Psi \times \right)\left(v^{n}+\delta v^{n}\right)\\ & \approx C_{b}^{l}C_{n}^{b}v^{n}+C_{b}^{l}C_{n}^{b}\delta v^{n}-C_{b}^{l}C_{n}^{b}\left(v^{n}\times \right)\Psi \\ & \approx v^{l}+C_{b}^{l}C_{n}^{b}\delta v^{n}-C_{b}^{l}C_{n}^{b}\left(v^{n}\times \right)\Psi \end{array}$$

where $v^{l}=C_{b}^{l}C_{n}^{b}v^{n}$ represents the velocity vector in the *l*-frame, $C_{n}^{b}$ the transformation matrix from the *n*-frame to the *b*-frame, $C_{b}^{l}$ the transformation matrix from the *b*-frame to the *l*-frame (i.e., attitude misalignment between pedestrian frame and smartphone frame). We assume that pedestrians walk on a level plane (a stair-climbing scenario consists of many small level planes) in the indoor environment, so the roll and pitch misalignment are equal to roll and pitch. The heading misalignment has always been the focus of PDR algorithm research and will not be described in detail here; refer to [42] for details. Accordingly, the observation equation for velocity in the *l*-frame is given by:

(11) $$\begin{array}{ll} \delta z_{l} & ={\hat{v}}^{l}-{\tilde{v}}^{l}\\ & =C_{b}^{l}C_{n}^{b}\delta v^{n}-C_{b}^{l}C_{n}^{b}\left(v^{n}\times \right)\Psi -n_{v} \end{array}$$

We note that when a pedestrian holds a smartphone in a swinging hand, the real speed of the smartphone is not consistent with the pedestrian’s walking speed. Therefore, the above pseudo-speed constraints will not provide an accurate positioning result and can even reduce the performance. In this case, the relative displacement constraints will be more suitable than a pseudo-velocity constraint. We store the position of the previous step, combined with the real-time estimated step length and heading at the current step, and thus the two steps of the relative displacement increment are given as:

(12) $$r_{k}^{n}=r_{k-1}^{n}+SL\cdot \left[\begin{array}{c} \cos\left(\psi_{k}\right)\\ \sin\left(\psi_{k}\right) \end{array}\right]$$

where $r_{k-1}^{n}$ and $r_{k}^{n}$ represent the north and east position in the *n*-frame, and *k−1* and *k* represent the respective step counts. Accordingly, the observation equation for the relative displacement increment in the *n*-frame is given by:

(13) $$\delta z_{p}={\hat{p}}_{12}^{n}-{\tilde{r}}_{k}^{n}=\delta p_{12}^{n}-n_{p}$$

where $p_{12}^{n}$ represents the first two elements of the position vector in the *n*-frame. $\delta p_{12}^{n}$ represents the position error vector of the first two elements and $n_{p}$ the measurement noise.

#### 2.3.3. Gravity Vector Update

Regarding Earth gravity sensing ability, tri-accelerometers are usually used to calculate the absolute horizontal angle (i.e., roll and pitch) with sufficient accuracy in the absence of external acceleration. We use the difference between the normal value of the tri-accelerometer observation vector and Earth’s gravity to determine whether external acceleration exists. In most cases, for users holding a smartphone in an arbitrary position, there is a very large probability that the singularity problem will manifest when the pitch angle reaches ±90°. Therefore, in this paper, tri-accelerometer readings are used directly to update the navigation attitude angle instead of using the roll and pitch angle. The accelerometer measurement model is given by [26]:

(14) $$\begin{array}{ll} \delta f^{n} & ={\hat{C}}_{b}^{n}{\tilde{f}}^{b}-f^{n}\\ & =\left(I-\Psi \times \right)C_{b}^{n}\left(f^{b}+b_{f}+n_{m}\right)-f^{n}\\ & =\left[\left(C_{b}^{n}f^{b}\right)\times \right]\Psi +C_{b}^{n}b_{f}+C_{b}^{n}n_{f} \end{array}$$

where $f^{n}={\left[\begin{array}{ccc} 0 & 0 & -g \end{array}\right]}^{T}$ represents the Earth gravity vector, ${\tilde{f}}^{b}$ the accelerometer reading vector, $b_{f}$ the accelerometer bias vector, and $n_{f}$ the measurement noise.

#### 2.3.4. Magnetic Field Vector Update

Tri-magnetometers are usually used to calculate the absolute heading in the absence of any external magnetic field interference (such as in an outdoor open environment) [51]. After compensating the magnetic declination, a high-precision absolute heading of 2° can be obtained. However, since frequent magnetic field disturbances are caused by building structures and metallic iron in indoor environments, the absolute heading calculated from the magnetometer reading is unreliable. To eliminate the error caused by magnetic field disturbances, many researchers have proposed using the quasi-static magnetic field (i.e., good stability in a small local area) to provide a relative heading change measurement (see [26] for details of quasi-static magnetic field period detection). Moreover, to avoid the influence of horizontal angle error, the magnetometer readings are used to update the navigation state by means of tight coupling. The magnetometer measurement model is given by [26]:

(15) $$\begin{array}{ll} \delta m^{n} & ={\hat{C}}_{b}^{n}{\tilde{m}}^{b}-m^{n}\\ & =\left(I-\Psi \times \right)C_{b}^{n}\left(m^{b}+n_{m}\right)-m^{n}\\ & =\left[\left(C_{b}^{n}m^{b}\right)\times \right]\Psi +C_{b}^{n}n_{m} \end{array}$$

where ${\tilde{m}}^{b}$ represents the magnetometer reading vector, ${\hat{m}}^{n}$ the calibration magnetic field of the quasi static magnetic field, and $n_{m}$ the measurement noise.

## 3. Results

In general, a pedestrian’s walking heading is the main factor influencing the accuracy of position estimation after training a gait model for the PDR algorithms. Thus, E-PDR always exhibits better navigation performance than normal PDR owing to its excellent heading accuracy, which is achieved by fusing the data from tri-gyroscopes, tri-accelerometers, and tri-magnetometers. Tri-accelerometers provide precise leveling angles in the absence of external acceleration, and tri-magnetometers provide relative heading change constraints to realize heading estimation when a pedestrian is walking in a quasi-static magnetic field. However, the existing tri-accelerometer and tri-magnetometer bias in a low-grade IMU will significantly degrade the navigation performance of E-PDR. In the work described this paper, an in situ hand calibration method was used to calibrate the tri-accelerometer bias [52] and an ellipsoid fitting method of [53] was employed to calibrate the tri-magnetometer bias before evaluating the navigation performance.

**Figure 3:**
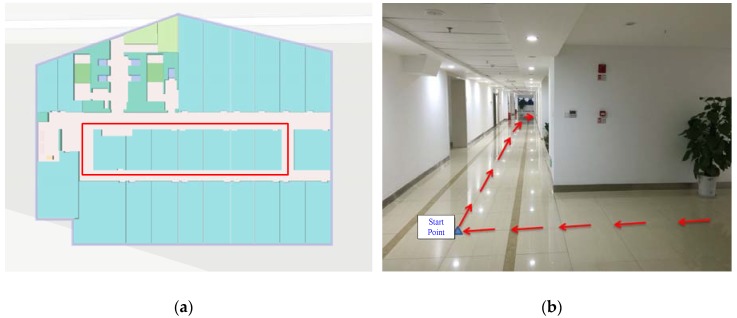
Trajectory of experiment. (**a**) Trajectory profile; (**b**) Experimental environment.

**Figure 4:**
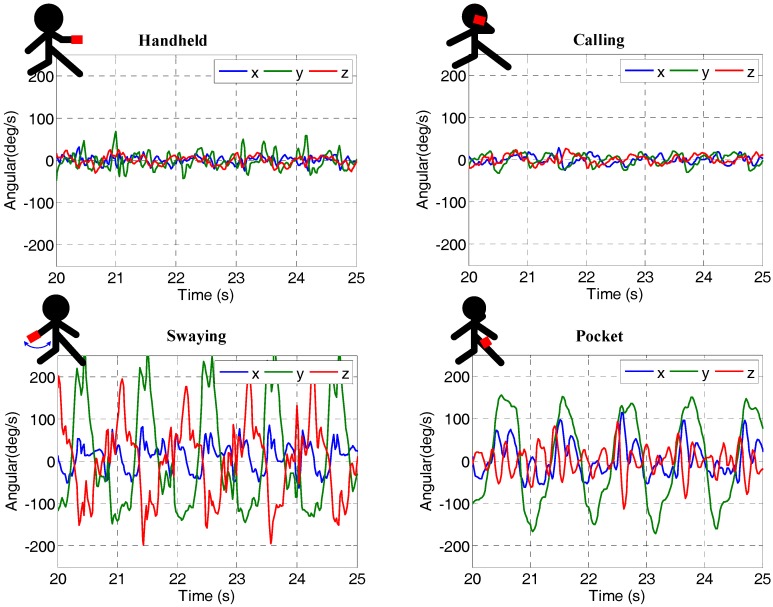
Four basic smartphone-holding positions and corresponding gyro readings.

Several indoor walking experiments were conducted using two phones (a Samsung S6 and a Xiaomi 4) to collect data. The two smartphones were held by different pedestrians following the same pre-planned path (approximately 80-m) three times, as shown by the red line in Figure 3. The Samsung S6 data is used as a sample to illustrate the experimental results. The tested pedestrian phone-holding positions included handheld (e.g., texting), calling (e.g., holding the phone close to an ear), swaying (e.g., walking while swinging the phone in the hand), and pocketing (e.g., keeping the phone in a front pants pocket). Figure 4 illustrates the tested phone motion modes and the corresponding gyro readings. In order to provide the reference values, we recorded the timestamps when passing a corner and wrote them into a file during the test. We also obtained the coordinates of the aforementioned corners by selecting the corresponding point on a high-precision map. Next, the coordinates of the line segments between the two corners were calculated by linear interpolation in time.

### 3.1. Position Estimation Performance Analysis for INS-Based Method

**Figure 5:**
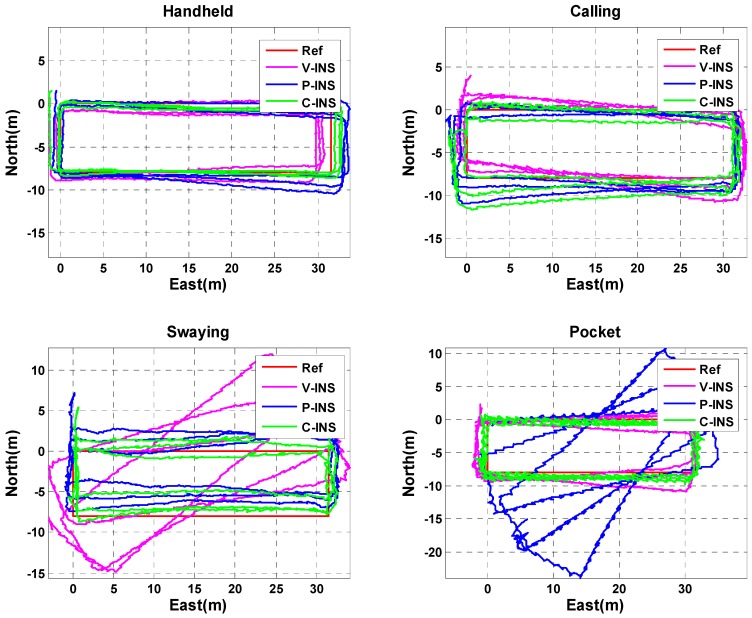
Trajectories of reference, V-INS, P-INS, and C-INS solutions for four different smartphone positions.

In this section, we analyze the navigation performance of the C-INS method in the four basic phone-motion modes described above. In [15,24,47], the pseudo-velocity from the gait model was employed for velocity updates to improve the INS velocity, and is called V-INS. In Ref. [48], the pseudo-position from E-PDR was used for position updates to improve INS position, and is called P-INS. In Figure 5, the red, magenta, blue, and green lines represent the reference trajectory, V-INS trajectory, P-INS trajectory, and C-INS trajectory, respectively. We can see from the figure that C-INS exhibits a robust position estimation performance in the four basic smartphone positions. V-INS suffered large position drift in the swaying position because the relative position between the smartphone and pedestrian’s body is always changing during one step period. The velocity of the smartphone is seriously inconsistent with the pedestrian’s walking speed, so the assumption of a velocity update is violated. The difference is that P-INS suffered a large position drift when the smartphone was put into a pocket, the reason is that the position calculated by the E-PDR alternately represents the position of the left and right feet. In other words, if the smartphone is put into the right pants pocket, the estimated position of E-PDR represents the position of the left leg when swinging the left leg.

### 3.2. Heading Estimation between C-INS and E-PDR Methods

**Figure 6:**
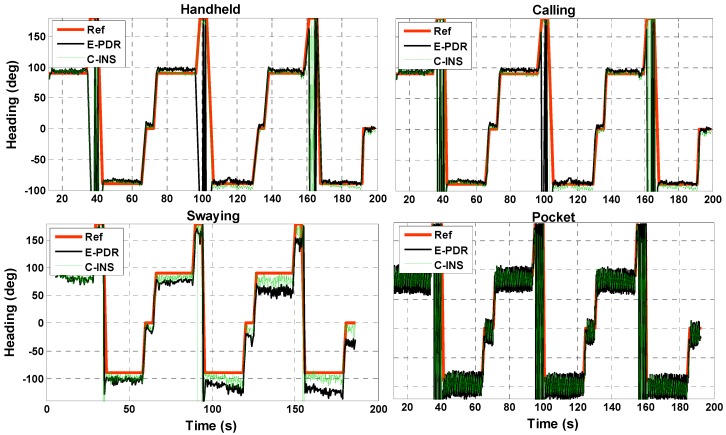
Heading of reference, E-PDR, and C-INS solutions for four different phone-holding positions.

**Figure 7:**
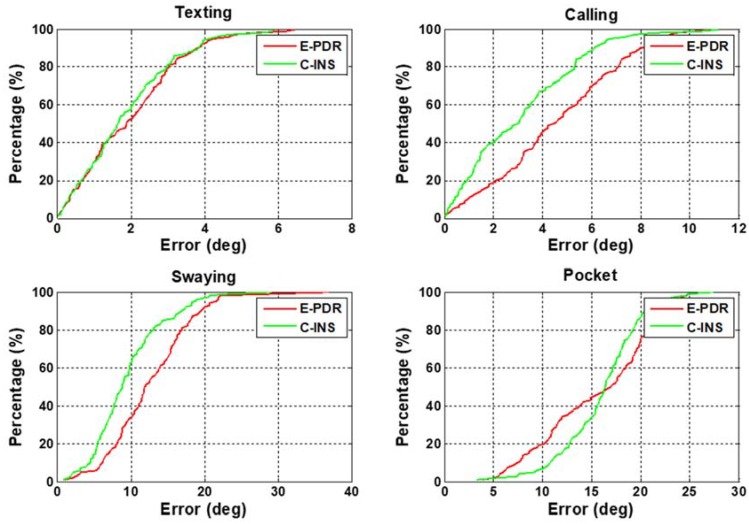
Heading angle cumulative error percentages of E-PDR and C-INS solutions for four different phone-holding positions.

In this section, we analyze heading performance to illustrate that C-INS exhibits consistent navigation performance with the E-PDR method. Figure 6 shows the heading angle result with four phone-holding positions calculated using the C-INS and E-PDR solutions. The red, green, and black solid lines represent the reference heading, heading estimation result of the C-INS method, and the heading estimation result of the E-PDR method, respectively. The corresponding heading cumulative error percentages are shown in Figure 7. As Figure 6 and Figure 7 show, the C-INS and E-PDR methods have similar heading performance when the smartphone is held in a hand or placed in a pocket. Moreover, the C-INS method can provide a higher precision of heading estimation in the positions of calling and swaying, because the external periodic acceleration caused by human activity reduces the chances of the accelerometer being used to correct the horizontal angle. Unlike the E-PDR method, the C-INS method uses a gait model to provide the relative position displacement or moving velocity, which is independent of the external horizontal acceleration. This results in the C-INS method being able to obtain a better heading estimation. The statistical heading performance of both algorithms in four phone-holding positions are illustrated in Table 1. As can be seen from the table, the root-mean-square (RMS) heading errors of the E-PDR method for the four different phone-holding positions are 2.4°, 5.3°, 14.0°, and 16.6°, while those for the C-INS method are 2.3°, 3.9°, 10.9°, and 16.6°, respectively.

### 3.3. Position Estimation between C-INS and E-PDR Methods

**Figure 8:**
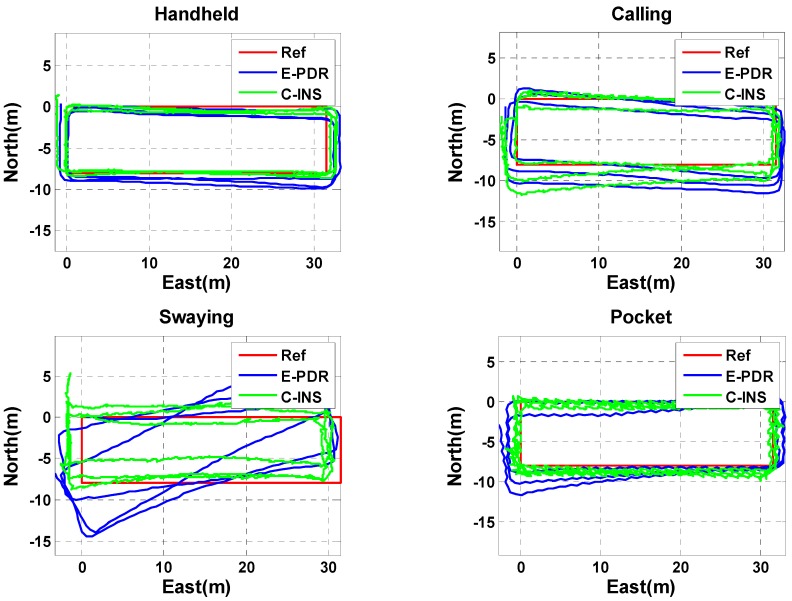
Trajectories of reference, E-PDR, and C-INS solutions for four different phone-holding positions.

**Figure 9:**
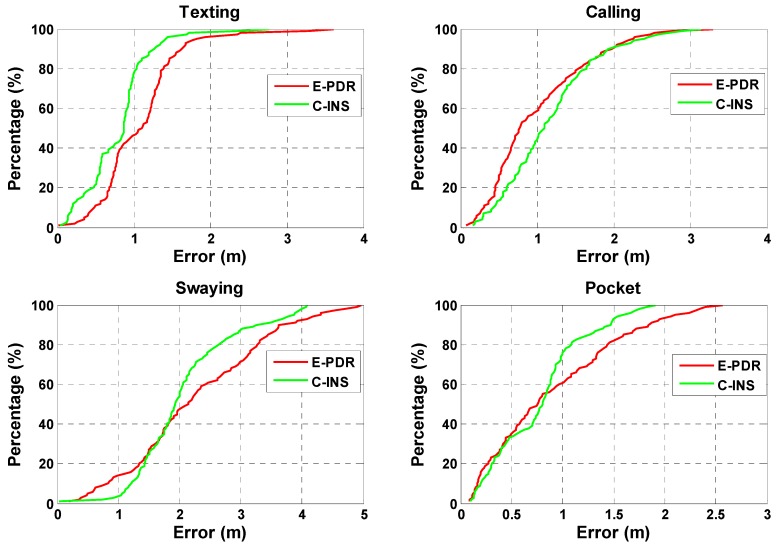
Position cumulative error percentages of E-PDR and C-INS solutions for four different phone-holding positions.

The position estimation results of the C-INS and E-PDR solutions in the experiment for the four basic phone-holding positions are shown in Figure 8. The red, green, and blue solid lines represent the reference trajectory, position result of the C-INS method, and position result of the E-PDR method, respectively. Figure 9 shows the position cumulative error percentages of the C-INS and E-PDR methods. As shown in Figure 8 and Figure 9, the position results calculated by the C-INS method have the same performance in the handheld, calling, and pocket positions, and even higher accuracy in the position of swaying, compared with the E-PDR method. The statistical positioning errors of both algorithms are illustrated in Table 2, the RMS position error of the E-PDR method for the four different positions (i.e., handheld, calling, swaying, and pocket) are 1.2, 1.2, 2.5, and 1.1 m, respectively, and those for the C-INS method are 0.9, 1.3, 2.2, and 0.9 m, respectively. Based on the same step model, the accuracy improvement of the heading angle cannot be completely reflected in the position error. Because the relative position of the phone and the human body is not fixed, the speed constraints and relative position constraints extracted from the step model sometimes do not conform to the actual situation.

### 3.4. Step Detection Failure between C-INS and E-PDR Methods

Based on the above description, the C-INS method can provide the same stable navigation performance (i.e., heading and position) as the E-PDR method, and also retain the basic property of SINS of autonomous propagation of pedestrian position by integrating acceleration and gyroscope data in the absence of training the step model. Therefore, the C-INS method will have the potential to change the current situation in the SINS-based method in which the mobile device cannot be applied in the field of pedestrian navigation for quick position error accumulation. In recent years, the performance of IMU built-in mobile equipment in the existing market has been greatly improved, so the C-INS method has the ability to maintain its navigation performance for several seconds when step detection is unavailable. However, step missing will greatly degrade the navigation performance of the E-PDR method because the E-PDR method only updates positions when a step event is successfully detected.

**Figure 10:**
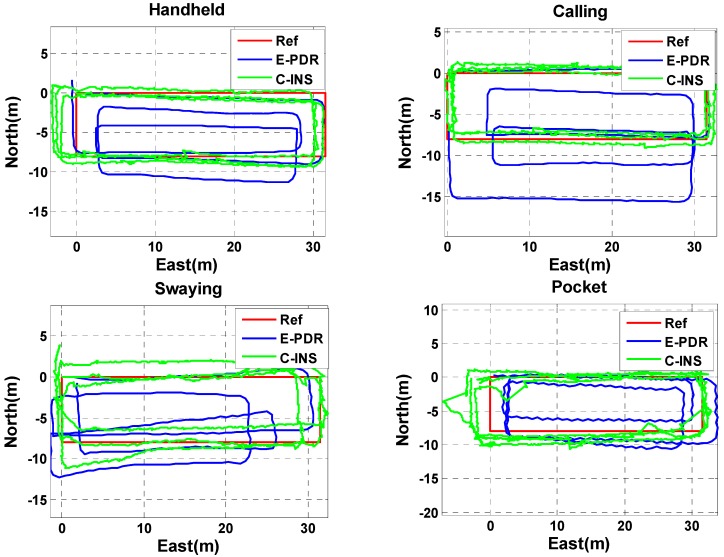
Trajectories of reference, E-PDR, and C-INS solutions with missing steps event for four different phone-holding positions.

To explore this issue, we designed an experiment in which the missing of step events was manually set to investigate the autonomic position estimation ability of the C-INS method. We set a period of 13 s, and found that step detection failed in the first 3 s (approximately five steps), and that a step event was detected successfully in the next 10 s. The gait model was used to update navigation state, for a total of 10 cycles. As the position result shows in Figure 10, the C-INS method exhibits a slight decrease in position accuracy when missing a small number of steps in the four basic phone-holding positions. However, the position result calculated by the E-PDR method is very sensitive to the step missing. If a pedestrian is walking at a normal velocity (i.e., 1.0–1.5 m/s), a 3-s period will cause a position error of approximately 3.5 m (a step length of 0.7 m).

**Figure 11:**
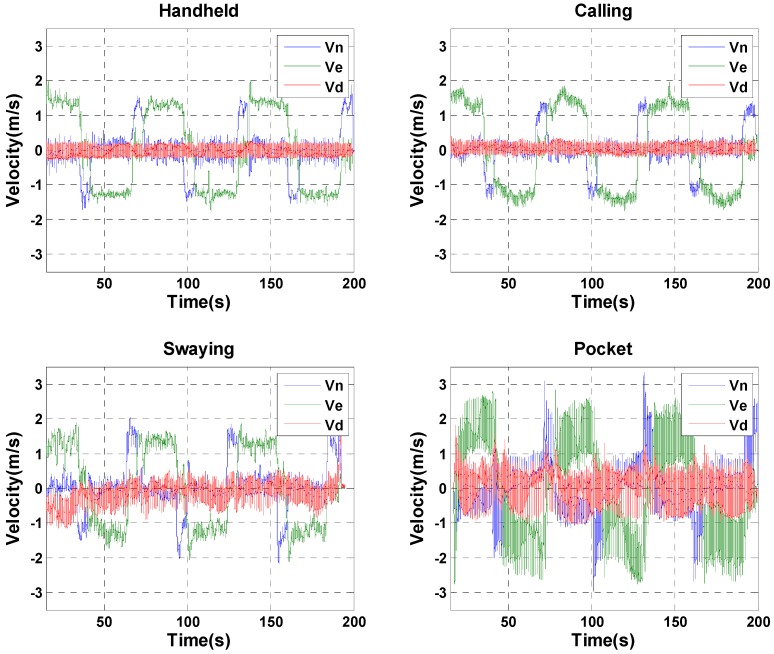
North, east, and down velocities of C-INS solution for four different phone-holding positions.

In addition to self-position estimation capability, the C-INS method can provide high-frequency position results (e.g., 50 Hz) that are the same as the data-sampling rate compared with the E-PDR solutions (i.e., 1–2 Hz). The high-frequency 3D velocity is also available for the C-INS method. As shown in Figure 11, the blue, green, and red solid lines represent the north, east, and down velocities, respectively. Figure 11 shows that the calculated velocity values are all at 1–2 m/s in the handheld, calling, and swaying positions, which are reasonable estimations for pedestrian walking velocity. In the pocket position, the velocity fluctuation is very large because each leg movement will cause a great acceleration of the smartphone. In this case, the average speed during a step circle is equal to the pedestrian’s walking speed.

## 4. Conclusions and Future Work

In this paper, we provide an advanced C-INS method using the built-in sensors of portable consumer devices. The C-INS method employs a gait model and motion constraints to provide pseudo-measurement (i.e., 3D velocity and 2D position increment) instead of using only pseudo-velocity measurements to realize a robust position estimation algorithm. In several real walking tests, we analyze the navigation performance differences between the E-PDR and C-INS method from three aspects: heading estimation, position estimation, and step detection failure. The C-INS method exhibits better performance in heading estimation and position estimation than the E-PDR method, because the C-INS method uses a gait model to improve the accuracy of roll and pitch angle, which is immune to the effects of external acceleration caused by personal daily activity. Moreover, the C-INS method has the ability to maintain position estimation in case of step detection failure, which is the nature of INS autonomous calculation.

It is foreseeable that mobile phone ownership will grow at an unprecedented rate. The quality of the built-in smartphone sensors are also expected to be greatly improved. Under these circumstances, the unsolved problem of smartphone-based PDR (i.e., misalignment angle estimation) may be well resolved by the powerful self-calculation ability of the C-INS method. Currently, the C-INS method can provide higher-frequency (e.g., 50 Hz) output, more abundant information (e.g., 3D velocity and height), and more robust heading and position estimation than the E-PDR method.

In future work, we plan to test and verify which grade of IMU will support the C-INS method to fix the issue of misalignment angle estimation. And more complex navigation scenarios will be used to evaluate the performance of C-INS, including movement between floors, elevators, opening/closing doors and changes in walking speed. In addition, some other absolute position measurements or distance measurements (e.g., UWB, vision, Wi-Fi, and BLE) will be used to control the position drift of C-INS.

## Figures and Tables

**Table 1:** Heading angle error of E-PDR and C-INS solution for four different phone-holding positions (units of degree).

	Handled		Calling		Swaying		Pocket	
	E-PDR	C-INS	E-PDR	C-INS	E-PDR	C-INS	E-PDR	C-INS
**Mean**	2.0	1.9	4.6	3.1	12.8	9.7	15.6	16.1
**RMS**	2.4	2.3	5.3	3.9	14.0	10.9	16.6	16.6
**Max**	6.4	5.8	10.8	11.1	36.8	29.1	27.4	27.4

**Table 2:** Position errors of E-PDR and C-INS solutions for four different phone-holding positions (units of m).

	Handled		Calling		Swaying		Pocket	
	E-PDR	C-INS	E-PDR	C-INS	E-PDR	C-INS	E-PDR	C-INS
**Mean**	1.09	0.81	1.00	1.16	2.28	2.08	0.90	0.77
**RMS**	1.22	0.92	1.20	1.33	2.54	2.23	1.11	0.90
**Max**	3.61	2.76	3.30	3.14	4.96	4.08	2.56	1.91

## References

[1] Alletto S., Cucchiara R., Del Fiore G., Mainetti L., Mighali V., Patrono L., Serra G. (2016). An indoor location-aware system for an IoT-based smart museum. IEEE Internet Things J..

[2] Li Y., Zhuang Y., Lan H., Zhang P., Niu X., El-Sheimy N. (2015). WiFi-Aided Magnetic Matching for Indoor Navigation with Consumer Portable Devices. Micromachines.

[3] Chen L.H., Wu E.H.K., Jin M.H., Chen G.H. (2014). Intelligent Fusion of Wi-Fi and Inertial Sensor-Based Positioning Systems for Indoor Pedestrian Navigation. IEEE Sens. J..

[4] Yang J., Wang Z., Zhang X. (2015). An iBeacon-based indoor positioning systems for hospitals. Int. J. Smart Home.

[5] Lin X.Y., Ho T.W., Fang C.C., Yen Z.S., Yang B.J., Lai F. A mobile indoor positioning system based on iBeacon technology. Proceedings of the International Conference of the IEEE Engineering in Medicine and Biology Society.

[6] Bekkali A., Sanson H., Matsumoto M. RFID Indoor Positioning Based on Probabilistic RFID Map and Kalman Filtering. Proceedings of the Third IEEE International Conference on Wireless and Mobile Computing, Networking and Communications (WiMOB 2007).

[7] Ruiz A.R.J., Granja F.S., Honorato J.C.P., Rosas J.I.G. (2011). Accurate Pedestrian Indoor Navigation by Tightly Coupling Foot-Mounted IMU and RFID Measurements. IEEE Trans. Instrum. Meas..

[8] Ozdenizci B., Coskun V., Ok K. (2015). NFC Internal: An Indoor Navigation System. Sensors.

[9] Yan J. (2010). Algorithms for Indoor Positioning Systems Using Ultra-Wideband Signals. Ph.D. Thesis.

[10] Angermann M., Frassl M., Doniec M., Julian B.J., Robertson P. Characterization of the indoor magnetic field for applications in Localization and Mapping. Proceedings of the International Conference on Indoor Positioning and Indoor Navigation.

[11] Fan X., Wu J., Long C., Zhu Y. Accurate and Low-cost Mobile Indoor Localization with 2-D Magnetic Fingerprints. Proceedings of the First ACM Workshop on Mobile Crowdsensing Systems and Applications.

[12] Pratama A.R., Widyawan, Hidayat R. Smartphone-based Pedestrian Dead Reckoning as an indoor positioning system. Proceedings of the International Conference on System Engineering and Technology.

[13] Yang Z., Wu C., Zhou Z., Zhang X., Wang X., Liu Y. (2015). Mobility Increases Localizability: A Survey on Wireless Indoor Localization using Inertial Sensors. ACM Comput. Surv..

[14] Mendoza-Silva G., Richter P., Torres-Sospedra J., Lohan E., Huerta J. (2018). Long-Term WiFi Fingerprinting Dataset for Research on Robust Indoor Positioning. Date.

[15] Zhuang Y., El-Sheimy N. (2015). Tightly-Coupled Integration of WiFi and MEMS Sensors on Handheld Devices for Indoor Pedestrian Navigation. IEEE Sens. J..

[16] Jimenez A.R., Seco F., Prieto C., Guevara J. A comparison of Pedestrian Dead-Reckoning algorithms using a low-cost MEMS IMU. Proceedings of the IEEE International Symposium on Intelligent Signal Processing.

[17] Davidson P., Piche R. (2017). A Survey of Selected Indoor Positioning Methods for Smartphones. IEEE Commun. Surv. Tutor..

[18] Qian J., Ma J., Ying R., Liu P., Pei L. An improved indoor localization method using smartphone inertial sensors. Proceedings of the International Conference on Indoor Positioning and Indoor Navigation.

[19] Goyal P., Ribeiro V.J., Saran H., Kumar A. Strap-down Pedestrian Dead-Reckoning system. Proceedings of the International Conference on Indoor Positioning and Indoor Navigation.

[20] Rai A., Chintalapudi K.K., Padmanabhan V.N., Sen R. Zee: Zero-effort crowdsourcing for indoor localization. Proceedings of the 18th Annual International Conference on Mobile Computing and Networking.

[21] Rong L., Zhiguo D., Jianzhong Z., Ming L. Identification of Individual Walking Patterns Using Gait Acceleration. Proceedings of the International Conference on Bioinformatics and Biomedical Engineering.

[22] Brajdic A., Harle R. Walk detection and step counting on unconstrained smartphones. Proceedings of the ACM International Joint Conference on Pervasive and Ubiquitous Computing.

[23] Diaz E.M., Gonzalez A.L.M., Müller F.D.P. Standalone inertial pocket navigation system. Proceedings of the 2014 IEEE/ION Position, Location and Navigation Symposium (PLANS 2014).

[24] Zhuang Y., Lan H., Li Y., El-Sheimy N. (2015). PDR/INS/WiFi Integration Based on Handheld Devices for Indoor Pedestrian Navigation. Micromachines.

[25] Rosario M.B.D., Lovell N.H., Redmond S.J. (2016). Quaternion-Based Complementary Filter for Attitude Determination of a Smartphone. IEEE Sens. J..

[26] Li Y., Lan H., Zhuang Y., Zhang P., Niu X., El-Sheimy N. Real-time Attitude Tracking of Mobile Devices. Proceedings of the International Conference on Indoor Positioning and Indoor Navigation.

[27] Yadav N., Bleakley C. (2014). Accurate orientation estimation using AHRS under conditions of magnetic distortion. Sensors.

[28] Afzal M.H. (2011). Use of Earth’s Magnetic Field for Pedestrian Navigation. Ph.D. Thesis.

[29] Renaudin V., Combettes C. (2014). Magnetic, Acceleration Fields and Gyroscope Quaternion (MAGYQ)-based attitude estimation with smartphone sensors for indoor pedestrian navigation. Sensors.

[30] Renaudin V., Afzal M.H., Lachapelle G. (2012). Magnetic perturbations detection and heading estimation using magnetometers. J. Locat. Based Serv..

[31] Lee J.K., Park E.J., Robinovitch S.N. (2012). Estimation of Attitude and External Acceleration Using Inertial Sensor Measurement during Various Dynamic Conditions. IEEE Trans. Instrum. Meas..

[32] Afzal M.H., Renaudin V., Lachapelle G. (2011). Use of Earth’s magnetic field for mitigating gyroscope errors regardless of magnetic perturbation. Sensors.

[33] Foxlin E. (2005). Pedestrian tracking with shoe-mounted inertial sensors. IEEE Comput. Graph. Appl..

[34] Rajagopal S. (2008). Personal Dead Reckoning System with Shoe Mounted Inertial Sensors. Master’s Thesis.

[35] Borenstein J., Ojeda L., Kwanmuang S. (2008). Heuristic reduction of gyro drift in IMU-based personnel tracking systems. J. Navig..

[36] Jimenez A.R., Seco F., Zampella F., Prieto J.C., Guevara J. Improved Heuristic Drift Elimination (iHDE) for pedestrian navigation in complex buildings. Proceedings of the International Conference on Indoor Positioning and Indoor Navigation.

[37] Zampella F., Khider M., Robertson P., Jiménez A. Unscented Kalman filter and Magnetic Angular Rate Update (MARU) for an improved Pedestrian Dead-Reckoning. Proceedings of the Position Location and Navigation Symposium.

[38] Gu Y. (2014). Foot-mounted Pedestrian Navigation based on Particle Filter with an Adaptive Weight Updating Strategy. J. Navig..

[39] Wahlström J., Skog I., Händel P. (2016). Smartphone-Based Vehicle Telematics: A Ten-Year Anniversary. IEEE Trans. Intell. Transp. Syst..

[40] Ladetto Q., Merminod B. Digital magnetic compass and gyroscope integration for pedestrian navigation. Proceedings of the 9th International Conference on Integrated Navigation Systems.

[41] Deng Z.A. (2015). Heading Estimation for Indoor Pedestrian Navigation Using a Smartphone in the Pocket. Sensors.

[42] Combettes C., Renaudin V. Comparison of misalignment estimation techniques between handheld device and walking directions. Proceedings of the International Conference on Indoor Positioning and Indoor Navigation.

[43] Kunze K., Lukowicz P., Partridge K., Begole B. Which Way Am I Facing: Inferring Horizontal Device Orientation from an Accelerometer Signal. Proceedings of the International Symposium on Wearable Computers.

[44] Yang X., Huang B., Miao Q. A step-wise algorithm for heading estimation via a smartphone. Proceedings of the Control and Decision Conference.

[45] Liu D., Pei L., Qian J., Wang L., Liu P., Dong Z., Xie S., Wei W. A novel heading estimation algorithm for pedestrian using a smartphone without attitude constraints. Proceedings of the Fourth International Conference on Ubiquitous Positioning, Indoor Navigation and Location Based Services.

[46] Davidson P., Takala J. (2013). Algorithm for pedestrian navigation combining IMU measurements and gait models. Gyroscopy Navig..

[47] Lan H. (2016). Multiple Systems Integration for Pedestrian Indoor Navigation. Ph.D. Thesis.

[48] Lin T., Li L., Lachapelle G. Multiple sensors integration for pedestrian indoor navigation. Proceedings of the International Conference on Indoor Positioning and Indoor Navigation.

[49] Shin E.-H. (2005). Estimation Techniques for Low-Cost Inertial Navigation. Ph.D. Thesis.

[50] Skog I., Handel P., Nilsson J.-O., Rantakokko J. (2010). Zero-Velocity Detection—An Algorithm Evaluation. IEEE Trans. Biomed. Eng..

[51] Abdul Rahim K. (2012). Heading Drift Mitigation for Low-Cost Inertial Pedestrian Navigation. Ph.D. Thesis.

[52] Li Y., Niu X., Zhang Q., Zhang H., Shi C. (2012). An in situ hand calibration method using a pseudo-observation scheme for low-end inertial measurement units. Meas. Sci. Technol..

[53] Tabatabaei S., Gluhak A., Tafazolli R. (2013). A Fast Calibration Method for Triaxial Magnetometers. IEEE Trans. Instrum. Meas..

